# Cognitive Control/Flexibility, Social Isolation, and Intrinsic Job Satisfaction of Intensive Care Unit Nurses

**DOI:** 10.3390/bs14070605

**Published:** 2024-07-17

**Authors:** Fatima Zehra Allahverdi, Nukhet Bayer

**Affiliations:** 1 Department of Psychology, Ankara Sosyal Bilimler Universitesi, 06050 Ankara, Türkiye; 2 Department of Nursing Management, Lokman Hekim University, 06530 Ankara, Türkiye; nukhet.bayer@lokmanhekim.edu.tr

**Keywords:** cognitive control, cognitive flexibility, social isolation, job satisfaction, intensive care unit nurses

## Abstract

Although cognitive control and flexibility have been examined in the past, this study examines their relationship in a stressful working environment, focusing on intrinsic job satisfaction using cognitive behavioral theory as a framework. This study examined cognitive factors (cognitive control and cognitive flexibility) and emotional state (intrinsic job satisfaction) while assessing the mediating role of social isolation, an external work environment variable. The study focused on intrinsic job satisfaction with extrinsic job satisfaction as a covariate. A cross-sectional questionnaire method was used. Two hundred and ten nurses from twelve intensive care units participated. Model one examined cognitive control while model two examined cognitive flexibility, accounting for 32% and 38% of the variance in intrinsic job satisfaction, respectively. Model one accounted for 13% of the variance in social isolation through cognitive control and extrinsic job satisfaction while model two accounted for approximately 14.91% of the variance in social isolation through cognitive flexibility and extrinsic job satisfaction. Combining the two models accounted for 17% of the variance in social isolation and 37.4% of the variation in intrinsic job satisfaction. The results emphasize the importance of training nurses in cognitive control and flexibility to increase intrinsic job satisfaction.

## 1. Introduction

Nurses working in the intensive care unit may encounter excessive workloads, situations requiring emergency intervention, patient deaths, conflicts with other employees, or unexpected problems due to the intensive interventions given to the patients [1] which can lead to low job satisfaction [2]. Excessive workload has been associated with lower nurse performance [3] and the intention to leave work [4]. Therefore, intensive care nurses should be ready to meet the unforeseen and constantly changing challenging job demands [5].

Despite the investigation of many variables that are related to actively dealing with the challenging job environments of nurses, there are many gaps in the literature. Research reveals two factors known to influence job performance: cognitive control and cognitive flexibility. “Cognitive flexibility is considered an executive function that allows individuals to adapt or switch what they are thinking and engage more readily in activities like reappraisal” [6] (p. 83). Cognitive control “encompasses a variety of top-down processes that enable us to direct our thoughts and behavior in accordance with current goals and environmental demands, and that form the basis for controlled processing of information” [7] (p. 1).Cognitive control and flexibility facilitate “goal-directed behaviors”, decision-making, problem-solving [8] (p. 1), and increase one’s productivity.

Although there has not been much research on cognitive control and cognitive flexibility, previous research has found the internal locus of control to be related to success, and researchers have attributed the results to the ability of nurses with internal control to “overcome their work environment” [9] (p. 173). The internal locus of control has been related to internal motivation [10] and job satisfaction [11] while the external locus of control has been related to burnout in nurses [12]. Examining cognitive control and cognitive flexibility is crucial since there has been much research on the level of nurse burnout [13] and its relationship with nursing errors [14]. Determining skills that can increase performance, increase job satisfaction, and reduce burnout is important.

Many studies have examined the relationship between unfavorable variables such as “maladaptive emotion regulation”, repetitive negative thinking [15] (p. 1200), and stress [16] on cognitive control. Some have examined the relationship of both cognitive control and flexibility on anxiety scores [17]. Additionally, there are studies that have examined the relationship of positive variables such as career adaptability with cognitive control and cognitive flexibility [18], and the psychological adjustment of nurses and cognitive flexibility [19].

To date, however, there is a lack of studies that have examined both cognitive control and flexibility together [7], especially with nurses. Additionally, although cognitive control and cognitive flexibility have been related to many positive variables [18,19]), studies that have examined their relationship with job satisfaction are minimal [9,20]. Job satisfaction, which is the feeling that expresses the level of content [6,21], can be described as a “pleasurable emotional state” [22] (p. 316).

Moreover, aside from job satisfaction, another issue related to the health, productivity, and efficiency of intensive care nurses is psychosocial risks [23]. The shortage of nurses and the excessive workload experienced can lead to feelings of isolation [24]. Social isolation is related to “perceptions of lack of availability of support and recognition, missed opportunities for informal interactions with co-workers, and not being part of the group” [25] (p. 196). Although social isolation has been studied extensively with the chronically ill and the elderly [26], this issue has not been addressed to the same extent with nurses. Therefore, the current study, guided by cognitive behavioral theory, selected a unique set of variables to contribute to the field. Below, the relationship of each variable to the selected theory is discussed.

### 1.1. Theoretical Framework

The theoretical framework selected for this analysis was cognitive behavioral theory. This theory was specifically selected for this study since cognitive behavioral theory examines the interconnection of cognitive, behavioral, and affective components that influence a person’s life. While previous theories were separated more broadly under behavioral and cognitive categories, cognitive behavioral theory combines the two perspectives and examines the “interactions between personal and environmental variables” [27] (p. 165). In this study, the focus is on the personal factors of cognitive control, cognitive flexibility, and internal job satisfaction, while the environmental variable is the social isolation of the nurses.

Although previous research has utilized cognitive behavioral theory to investigate the work environment, examining satisfaction, work ethic, career goals, and more [28], a gap still exists within the literature.

### 1.2. Cognitive Control and Cognitive Flexibility

Previous research has found cognitive flexibility in nurses to be related to happiness [20]. Similarly, a negative relationship was found between cognitive flexibility levels and despair levels [29]. It was further related to resilience [30]. Thus, cognitive flexibility was found to be related to affective components or emotions.

Cognitive flexibility is often regarded as an important component of CBT [31]. “Unfortunately, few researchers have sought to understand the concept of cognitive control… [therefore]…we know little about the” topic [32]. There are not sufficient studies that address both cognitive control and flexibility together [7] in nurses. Despite researchers relating cognitive flexibility directly with cognitive behavioral theory [33,34], examining cognitive control alongside cognitive flexibility is important since there are researchers that examine both variables and discuss the relevance of studying both at the same time [7,8].

Since cognitive variables influence affective components, and due to nurses having very low job satisfaction (affective component), the current research predicted that the higher the cognitive control and flexibility, the higher the job satisfaction would be. Although research on cognitive flexibility is mixed with some researchers finding no effect of cognitive flexibility on job satisfaction [35], and others finding a relationship between cognitive flexibility and medical program satisfaction [6], the current study predicted that the divergent results could be due to many researchers evaluating job satisfaction as a whole rather than examining intrinsic job satisfaction, or aspects of the job itself.

Among other nurse working units, previous research discusses how the critical care (intensive care) unit is especially stressful, and since cognitive control and cognitive flexibility are particularly important in stressful settings [8], the current study focuses on intensive care unit nurses.

### 1.3. Job Satisfaction

Job satisfaction can be divided into intrinsic and extrinsic components. Intrinsic satisfaction refers to how people feel related to aspects of the job itself and involves personal factors [36] such as task significance, skill variety, and challenge [37]. Extrinsic satisfaction is related to aspects “that are external to the job tasks or work itself” [38] such as promotions, bonuses, and working conditions [37].

As [39] states, “Although many studies have explored factors affecting the job satisfaction of nursing assistants (e.g., job stress, psychological empowerment, and received support from peers and managers, these studies did not consider the personal abilities of nursing assistants” (p. 1). Given that cognitive control and cognitive flexibility are both individual traits, examining its relationship with “personal traits, capabilities, knowledge level, and experiences” of job satisfaction, or intrinsic job satisfaction, is important [36] (p. 629). Therefore, in this study, the effect of outside factors on job satisfaction, extrinsic job satisfaction, was removed. With the high workloads and ever-changing job demands [5], assessing satisfaction with aspects related to the job (intrinsic factors) is vital.

Since low job satisfaction is a major concern within the field of nursing and has been related to negative consequences such as high turnover, burnout, medical errors, and poor patient care quality [40,41,42], studying cognitive factors (personal abilities) and their relationship with intrinsic job satisfaction is important.

### 1.4. Social Isolation

Nurses who are isolated have higher turnover intentions, experience higher burnout, feel lonelier, and experience lower job satisfaction [43]. Previous research found extrinsic factors such as wage increase or job rotation as less related to job satisfaction compared to a cooperative environment [44]. Social support was found to indirectly influence job satisfaction [45]. Researchers emphasize the need to conduct research on the feelings of isolation of nurses [24].

Ref. [46] discuss how isolation is “frequently encountered by CBT therapists” and refer to it as one of the four existential concerns (freedom, death, isolation, and meaninglessness) that have begun to be addressed in CBT (p. 209). Cognitive behavioral theory investigates both the internal and external worlds of an individual [47]. Additionally, according to cognitive behavioral theory, the “self, relationships, the world, and the future shape emotions and behaviors” [48].

Since cognitive control and cognitive flexibility are both cognitive factors, and internal job satisfaction is a feeling/emotion, social isolation—related to relationships and the job environment—was expected to influence the relationship between cognition and emotions. The social isolation experienced by nurses is a pertinent aspect linked to their working conditions, and institutions are not yet addressing how intensive care units can reduce the effects of work environment-related social isolation [49]. The paucity of published research in nursing about these concepts warrants a closer examination especially since social isolation was found to be related to “less organizational commitment and more turnover intentions” [24] (p. 209).

Based on previous findings and the utilized theory, the researchers hypothesized a negative relationship between the mediator social isolation and intrinsic job satisfaction. Similarly, the researchers hypothesized a negative relationship between cognitive factors and social isolation.

The research questions of the study were as follows:

1. What are the cognitive control, cognitive flexibility, social isolation, and job satisfaction (intrinsic and extrinsic) levels of intensive care nurses?
2. Does social isolation mediate the relationship between cognitive control, cognitive flexibility, and intrinsic job satisfaction in intensive care nurses with extrinsic job satisfaction as a covariate?

## 2. Methodology

### 2.1. Design

This study utilized descriptive and cross-sectional data that were collected between September and December of 2022. The study used a mediation model.

### 2.2. Sample

The study consisted of nurses working in 12 different intensive care units of different public hospitals in the capital city of Turkey. The G*Power 3.1 program was used to calculate the power of the study. According to the calculations, to exceed the 95% value in determining the power of the study, a total of 107 people were needed at a 5% significance level and an effect size of 0.092 (df = 104; F = 3.08). Questionnaires were used to collect the data. A total of 323 nurses agreed to participate in the study. However, due to some participants not completing the survey completely, and leaving major portions blank, they were removed, resulting in 210 completed surveys. To participate in the study, the nurses had to have at least one year of experience working in the intensive care unit.

Collecting data from intensive care unit nurses is more difficult compared to other units. Although the response rate was not as high as aimed, it was higher/similar to other studies. Ref. [50] collected data from more than one hospital with one hospital providing a 54% response rate and another a 37% response rate. Ref. [51] examined a total of 19 studies on intensive care unit nurses, finding that only three of the studies were able to provide a response rate higher than 45%. Therefore, compared to the literature within this area of research, the response rate for the current study can be deemed acceptable.

### 2.3. Procedures

The researchers contacted the intensive care manager nurses of each of the hospitals, and the data collection forms were given to the manager nurses in a closed envelope which was then passed on to the intensive care unit nurses. Several days were given for the nurses to fill out the forms before they were collected from the nurse managers. Each participant confirmed that they voluntarily participated in the study. The data forms of the participants who gave incomplete answers were not included in the study.

The study was approved by the Non-Interventional Clinical Research Ethics Committee (Decision No: 20227143 and Code No: 2022131) on 21 September 2022. Written permission was obtained from the hospital administrations where the study was conducted. Nurses who were invited to the study were informed about the study, and those who gave their consent to volunteer were included in the study. Nurses were able to respond to the forms without providing any identifying information.

### 2.4. Data Collection Tools

The personal information form, Cognitive Control and Cognitive Flexibility Scale, Minnesota Job Satisfaction Scale, and social isolation scale were used to collect data. Information on the data collection tools is given below.

### 2.5. Personal Information Form

The personal information form asks questions related to biological sex, education, marital status, whether a person has kids, shift, position, number of years on the job, and age. The question on education asks whether the participant finished a health vocational high school, associate degree, bachelor’s degree, or graduate school. The shift question was asked to determine if participants were in a continuous day shift, continuous night shift, or mixed-shift work. The nurses also specified their positions: nurse supervisor, intensive care unit nurse, and manager nurse.

### 2.6. Minnesota Job Satisfaction Scale [38] 

The Job Satisfaction Scale was developed in 1967 by [38]. The Turkish adaptation of the scale was made by [52]. The scale uses a 5-point Likert scale. It consists of 20 items that determine intrinsic and extrinsic satisfaction factors. The highest score that can be obtained from the scale is 100, and the lowest score is 20. A score closer to 20 represents low satisfaction, while a score closer to 100 indicates high satisfaction. In this study, the Cronbach Alpha of the Minnesota Job Satisfaction Scale was α = 0.812.

### 2.7. Nottingham Health Profile (NHP)—Social Isolation [53]

The Nottingham Health Profile (NHP) is used to evaluate the quality of life. The current study used the social isolation scale which is one of the 6 subsections of the NHP questionnaire. Ref. [54] adapted the social isolation scale to Turkish. The scale consists of 5 questions and uses a 5-point Likert style. Higher scores represent an increase in perceived social isolation. In this study, the Cronbach Alpha of the social isolation scale was α = 0.842.

### 2.8. Cognitive Control and Flexibility Questionnaire (CCFQ; [8])

The scale was developed by [8] to assess whether individuals believe they can control negative unwanted thoughts and feelings and their ability to adapt to stressful situations flexibly. It was adapted into Turkish by [55]. There are a total of 18 items, with nine items on cognitive control and nine items on cognitive flexibility. The cognitive control sub-dimension had a Cronbach Alpha of α = 0.83 while the cognitive flexibility sub-dimension had a Cronbach Alpha of α = 0.93. A 7-point Likert scale is used.

## 3. Findings

As seen in Table 1, of the 210 nurses, 8 of them were nurse supervisors, 190 of them were intensive care unit nurses, and 12 of them were manager nurses. Most of the nurses were in the field between one to four years (80% of the participants) when surveyed ($\bar{x}$ = 4.04, sd = 4.13). Around 77% of the participants were single and 65.7% were female. Around 82% of the participants were between the ages of 23 and 28 ($\bar{x}$ = 27.25, sd = 4.48). Around 90% of the nurses worked in shifts, switching between mornings and evenings.

Table 2 provides the means and standard deviations, and Table 3 provides the correlations. As seen in Table 2, the nurses’ Cognitive Control and Cognitive Flexibility Scale (BPIQ) total dimension score was 4.75 ± 1.95, the cognitive control sub-dimension mean score was 4.34 ± 1.06, and the cognitive flexibility sub-dimension mean score was 5.10 ± 1.13. The mean score of the Minnesota Job Satisfaction Scale (MIS) was found to be 3.02 ± 0.56. The mean MIDO intrinsic job satisfaction sub-dimension score was 3.15 ± 0.63, and the extrinsic job satisfaction sub-dimension’s mean score was 2.83 ± 0.67. The mean score of the perceived social isolation scale (ASIS) was found to be 2.14 ± 0.82. Homoscedasticity, linearity, and multicollinearity were checked for the study. The results showed that the data were normally distributed.

### 3.1. Hayes Technique 

A mediation model was used utilizing the PROCESS macro to examine the relationship between nurses’ intrinsic job satisfaction, cognitive control level, and social isolation. Two hypotheses were tested. Two models were created since the PROCESS macro only accepts one independent variable per model [56].

### 3.2. First Hypothesis

**Figure 1:**
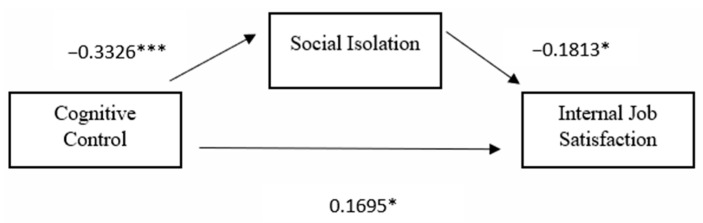
Standardized path coefficients for the proposed mediation model one, with external job satisfaction as a covariate *** *p* < 0.001, * *p* < 0.05.

For its first hypothesis, the study assessed the mediating role of social isolation on the relationship between cognitive control and intrinsic job satisfaction while controlling for extrinsic job satisfaction scores as can be seen in Figure 1. The results revealed a significant indirect impact of cognitive control on intrinsic job satisfaction through social isolation (β = 0.0354, t = 2.5286, *p* < 0.001). Cognitive control had a significant impact on social isolation (β = −0.255, t = −5.010, *p* < 0.001), indicating that as cognitive control decreased, social isolation increased. Similarly, as social isolation increased, intrinsic job satisfaction decreased (β = −0.1387, *p* < 0.05). Extrinsic job satisfaction was found to have an insignificant impact on social isolation. The model accounted for approximately 12.68% of the variance in social isolation through cognitive control and extrinsic job satisfaction. Social isolation partially mediated the relationship between cognitive control and intrinsic job satisfaction. The direct effect of cognitive control on intrinsic job satisfaction in the presence of the mediator was also significant (β = 0.0997, *p* < 0.05). Although extrinsic job satisfaction had an insignificant impact on social isolation, it was found to be a significant covariate affecting intrinsic job satisfaction (b = 0.445, t = 8.309, *p* < 0.001).

Thus, higher levels of cognitive control and extrinsic job satisfaction resulted in higher levels of intrinsic job satisfaction. This suggested cognitive control had a positive influence on intrinsic job satisfaction both directly and indirectly through its relationship with social isolation. For this model, 31.55% of the variance in intrinsic job satisfaction can be accounted for by the predictors. The mediation analysis and summary are presented in Table 4 and Table 5.

### 3.3. Second Hypothesis

**Figure 2:**
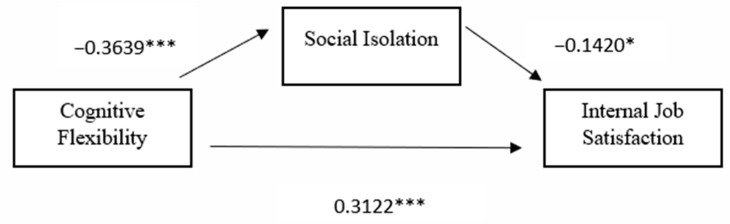
Standardized path coefficients for the proposed mediation model two, with external job satisfaction as a covariate *** *p* < 0.001, * *p* < 0.05.

For its second hypothesis, the study assessed the mediating role of social isolation on the relationship between cognitive flexibility and intrinsic job satisfaction while controlling for extrinsic job satisfaction scores as can be seen in Figure 2. The results revealed a significant indirect impact of cognitive flexibility on intrinsic job satisfaction through social isolation (b = 0.0287, t = 2.1103, *p* < 0.001). As cognitive flexibility increased, social isolation decreased (b = −0.2640, t = −5.6657, *p* < 0.001). Similarly, as social isolation increased, intrinsic job satisfaction decreased (β = −0.1087, *p* = 0.0181). Extrinsic job satisfaction was found to have an insignificant impact on social isolation. The model accounted for approximately 14.91% of the variance in social isolation through cognitive flexibility and extrinsic job satisfaction. Social isolation partially mediated the relationship between cognitive flexibility and intrinsic job satisfaction. The direct effect of cognitive flexibility on intrinsic job satisfaction in the presence of the mediator was also significant (b = 0.1447, *p* < 0.001). Additionally, although extrinsic job satisfaction had an insignificant impact on social isolation, it was found to be a significant covariate affecting intrinsic job satisfaction (b = 0.4509, t = 8.6463, *p* < 0.001).

Thus, higher levels of cognitive flexibility and extrinsic job satisfaction resulted in higher levels of intrinsic job satisfaction. This suggested cognitive flexibility had a positive influence on intrinsic job satisfaction both directly and indirectly through its relationship with social isolation. For this model, 37.74% of the variance in intrinsic job satisfaction can be accounted for by the predictors. The mediation analysis and summary are presented in Table 6 and Table 7.

### 3.4. Baron and Kenny Technique 

Following these analyses, the Baron and Kenny technique [57] was utilized (AMOS-SPSS) to examine the indirect effects using a mediation analysis with a bootstrap procedure (2000 samples) and bias-corrected bootstrap confidence interval (90%). The model provided a good fit (x2/df = 0.124, CFI = 0.99, TLI = 0.93, GFI= 0.99, RFI = 0.88, RMSEA = 0.08). In AMOS, both cognitive control and cognitive flexibility were included together in the model. The results indicated a partial mediation for social isolation on the effect of cognitive flexibility on intrinsic job satisfaction. For cognitive control, although the result approached significance for the total effect, it was not significant. However, full mediation was found on the effect of cognitive control on social isolation, and social isolation on intrinsic job satisfaction. The model accounted for 17% of the variance in social isolation and 37.4% of the variance in intrinsic job satisfaction. The mediation summary is presented in Table 8.

## 4. Discussion

The current study aimed to shed light on the relationship between cognitive factors (cognitive control and cognitive flexibility) and the affective component of intrinsic job satisfaction. The cognitive behavioral theory guided the study. Cognitive behavioral theory examines both the internal and external worlds [47]. Therefore, due to the impact of social isolation not being addressed sufficiently in nursing, and the profound impact of social isolation on the workplace atmosphere [43], the outside work environment of social isolation was examined. The excessive workload of intensive care unit nurses [1,58] can diminish times that allow for connection with colleagues [24], socially isolating employees.

Two statistical techniques were used to assess the relationship of the variables. The Haynes technique and the Baron and Kenny technique resulted in slightly different results. Utilizing the Haynes method, cognitive control and cognitive flexibility were both found to have a significant indirect effect on intrinsic job satisfaction through social isolation. Similarly, both were found to have a direct effect, thus supporting partial mediation. Accordingly, the cognitive behavioral theory was supported. As expected, findings indicated that the individual cognitive aspects related to engaging in reappraisal and directing thoughts towards goals predicted the feelings related to the job itself. Similarly, the outside or external aspect as referred to in CBT, social isolation, influenced the relationship of the variables. The results further support and augment the existing literature.

Similar to [24] results, demonstrating a relationship between social isolation and work well-being and professional commitment, the current study demonstrated a relationship between social isolation and intrinsic job satisfaction. Examining social isolation is critical since previous research demonstrates its relationship with introversion, loneliness, burnout, and depression [59].

Although previous research has demonstrated that social isolation is related to higher levels of hopelessness and job burnout [60], and that job burnout reduces job satisfaction [61,62], the current study made a direct link between social isolation and intrinsic job satisfaction.

The results indicated that the model accounted for 17% of the variance in social isolation and 37.4% of the variance in intrinsic job satisfaction. Examining a very thin slice of social isolation by looking at perception can be limiting and might explain why a larger percentage was not explained in the model. As [63] point out, “social isolation cannot be fully evaluated if a measurement only assesses specific aspects, such as social disconnectedness or perceived isolation” (p. 1143). Therefore, these researchers measured social isolation from several angles by looking at social support, the perception of isolation, social engagement, and social networks. They found that each 1% “increase in social isolation” was related to a 20–24% decrease in cognitive functioning. Therefore, defining social isolation and examining several angles can provide a more comprehensive result [63].

Social isolation has become a topic of research since the COVID-19 pandemic, with researchers supporting the use of cognitive behavioral therapy techniques to circumvent the negative impact of social isolation [64]. Ref. [64] emphasize the importance of taking on new challenges and discuss how social isolation is a newfound area of research that requires careful consideration and scrutiny.

Social isolation, which has been on the rise and has been shown to be “twice [as] harmful to physical and mental health as obesity”, is commonly treated with cognitive behavioral therapy [65] (p. 33). Similarly, the US Surgeon General “has expressed an urgency to address loneliness and social isolation because of its devastating effects” [66] (p. 1). Social isolation, dubbed as an existential crisis by some researchers [46] as an outside work environment, was found to be significant in this study, further emphasizing the importance of addressing social isolation within work environment settings. Thus, the current study points out the importance of considering outside work environment variables that may influence the relationship between cognitions and affective components.

Upon closer examination using the Baron and Kenny method, although social isolation partially mediated the relationship between cognitive flexibility and intrinsic job satisfaction, social isolation fully mediated the relationship between cognitive control and intrinsic job satisfaction. Although the percentage of variance was similar, cognitive flexibility scores were found to be higher compared to cognitive control.

Ref. [33] discuss how although many theories are referred to in the field of psychology, cognitive behavioral theory is among the most commonly utilized. However, there is a lack of specificity in defining the key aspects or concepts of each theory [67]. Cognitive flexibility, which is related to an ability to adapt to new situations, is considered by some researchers to involve affective, behavioral, and cognitive components all at once making it directly related to cognitive behavioral theory [33,34]. According to [68], referred to by [34], cognitive flexibility consists of “individuals’ being aware that there are various ways and alternative options, having a desire to adapt to new situations and be flexible, and believing that s/he has the competencies to be flexible” (p. 121). In fact, cognitive flexibility is one of the main goals of cognitive behavioral therapy. This could be one explanation for why cognitive flexibility was found to be significant while cognitive control was not found to be significant since it only addresses the cognitive aspect.

Similarly, ref. [15] discuss how cognitive control training by itself is insufficient for “adaptive emotion regulation” (p. 1199). Thus, although cognitive control and its training have been associated with a reduction in repetitive negative thinking (RNT), their effectiveness is minimal on their own when they are not used to augment a treatment or program. Consequently, it can be argued that cognitive control does not have a sufficient impact on its own similar to cognitive flexibility. This could possibly be due to it mostly focusing on the cognitive aspects, without incorporating the behavioral and affective components similar to cognitive flexibility. Additionally, although the “controlled processing of information” is integral to job performance and satisfaction, the ability to “adapt or switch” and participate in reappraisal can be more directly related to intrinsic job satisfaction [7] (p. 1).

Considering the necessity of intensive care nurses to have strong communication skills with patients, patient relatives, teammates, and other hospital staff while providing treatment care for the patients, a higher cognitive flexibility score compared to cognitive control can be considered normal. Another reason why cognitive flexibility might have appeared to be more related to the concepts studied is that individuals with high cognitive flexibility “adapt more easily to the different characteristics of their patients”, “collaborate more easily with the caregivers”, and “produce alternatives in the face of problems”, and can adapt more easily to the constantly “changing and developing nursing roles” [69] (p. 3). Individuals who have higher cognitive flexibility can better deal with stress and are less likely to exhibit “pathologic reactions” to stressful experiences [20]. High cognitive flexibility was found to be related to more carefully assessing options during decision-making [70].

The results of this study provide support for the idea that cognitive flexibility positively influences intrinsic job satisfaction (an affective component). Similarly, ref. [20] found cognitive flexibility to be positively related to happiness (feeling). Ref. [71] discusses how positive emotions are strongly related to cognitive flexibility. Thus, the findings of the current study align with [6] rather than [35] who did not find cognitive flexibility to influence job satisfaction.

Furthermore, some researchers argue that cognitive control is a very specific subcomponent of cognitive flexibility [19,20] while others examine both variables separately [8]. Other studies refer to how “cognitive flexibility has been identified as a component of cognitive control processes from a neuroscience perspective” [72] (p. 25). If the first perspective is taken, it might explain why cognitive flexibility was found to be more related to the variables examined compared to cognitive control. It could be argued that cognitive flexibility is a wider concept, thus allowing for more variability. More studies are necessary since cognitive control and cognitive flexibility comprise “two distinct, yet overlapping dimensions” [8] (p. 16).

The results of the current study further expressed the necessity of studying the job satisfaction of nurses. As expected, similar to other researchers [73,74,75] who found job satisfaction to generally be in the undecided and low range, the current study found job satisfaction scores to be low. Previous studies on nursing [73,74,75] examined all working units together while the current study added to the literature by focusing on intensive care unit nurses, a sub-group of nurses that are more difficult to collect data from.

Training nurses, especially in stressful working units, to increase their cognitive control and cognitive flexibility skills can alleviate some of the pressure felt in the work environment and can increase intrinsic job satisfaction. Additionally, workplaces can examine the structure of their buildings since previous research has found that even floor planning in hospitals and the design of the working units can influence the level of communication between employees [76]. Therefore, hospital infrastructures as well as the work schedules and designs can be examined with regard to social isolation. This is vital since previous studies along with the current study contribute to the field and discuss the importance of belongingness at work and job satisfaction [77].

## 5. Limitations

Due to the current study being cross-sectional in nature, although the relationship of the variables was discussed, a direct causal relationship could not be made. Additionally, the sample of the study was collected from the capital city. It is possible that there is more social isolation and more workload in the city compared to more rural areas. Therefore, the results of the study should be examined with caution due to issues with generalizability. Additionally, the specific sample of the study consisted of 77% single individuals with 82% of the participants being between the ages of 23 and 28. Hence, if the sample had consisted of older adults, the results might have been different.

## 6. Future Studies

Since there is a lack of studies on cognitive control and flexibility, especially regarding intensive care unit nurses, more research on this topic is necessary for comparison. It is especially relevant to collect data from intensive care unit nurses to determine the commonality of the findings of the current study [50,51] since this group is more difficult to collect data from. Moreover, other nursing units should also be compared to determine if any differences exist. Future studies can further examine other cognitive factors that might influence intrinsic job satisfaction (an affective component).

Since job burnout and social isolation are strongly related [60], which is also related to lower job satisfaction [61,62], future studies can incorporate burnout and stress levels to determine its dynamic relationship with cognitive control, cognitive flexibility, social isolation, and both intrinsic and extrinsic job satisfaction. Additionally, social isolation can be measured using not only the perception of isolation but also social support, social engagement, etc. In fact, utilizing a quasi-experimental approach can highlight the role of social isolation [63].

Additionally, since anxiety has been related to reductions in cognitive control and flexibility [17], future studies can utilize randomized controlled trials similar to [78] to determine if cognitive flexibility and control training could increase the resilience of nurses and thus increase their work and psychological adjustment.

Cognitive flexibility has been associated with a positive effect on professional autonomy [69]. In the future, the self-determination theory [79] can be utilized to assess social isolation, cognitive flexibility, and its relationship with autonomy and competence.

## Figures and Tables

**Table 1:** Distribution of nurses according to some sociodemographic characteristics.

Characteristics		*N*	(%)
Sex	Female	138	(65.7)
	Male	72	(34.3)
Education	Health Vocational High School	9	(4.3)
	Associate Degree	3	(1.4)
	Bachelor’s Degree	196	(93.3)
	Graduate Studies	2	(1.0)
Married Status	Married	48	(22.9)
	Single	162	(77.1)
Has Kids	Yes	29	(13.8)
	No	181	(86.2)
Working Style	Continuous Day Shift	19	(9.1)
	Continuous Night Shift	3	(1.4)
	Shift work	188	(89.5)
Number of Years on the Job	1–5	175	(83.3)
	6–10	20	(9.5)
	12–16	11	(5.2)
	18–26	4	(2)
Position	Nurse Supervisor	8	(3.8)
	Intensive Care Unit Nurses	190	(90.5)
	Manager Nurses	12	(5.7)
Age	22–30	183	(87.1)
	31–40	23	(11)
	41–50	4	(1.9)

**Table 2:** Descriptive statistics and scale mean scores of nurses.

Measures	$\bar{x}$	Sd	Minimum	Maximum
Cognitive Control and Flexibility Scale	4.751	0.949	1	7
Cognitive Control	4.399	1.064	1	7
Cognitive Flexibility	5.102	1.126	1	7
Minnesota Job Satisfaction Scale	3.026	0.560	1	4.45
Intrinsic Job Satisfaction	3.154	0.625	1.17	4.42
Extrinsic Job Satisfaction	2.834	0.665	1	6.75
Perceived Social Isolation	2.139	0.817	1	5

**Table 3:** Bivariate correlations.

	1	2	3	4
1. Intrinsic Job Satisfaction	1			
2. Cognitive Flexibility	0.341	1		
3. Cognitive Control	0.275 **	0.502 **	1	
4. Social Isolation	−0.301 **	−0.370 **	−0.342 **	1

** *p* < 0.01.

**Table 4:** Model one mediation analysis for cognitive control.

		M (Social Isolation)						Y (Life Satisfaction)		
Antecedent		B	SE	*p*	β		B	SE	*p*	β
X (Cognitive control)	a	−0.256	0.050	0.000	−0.333	c’	0.100	0.035	0.005	0.170
M (Social isolation)						b	−0.139	0.054	0.000	−0.181
		R^2^ = 0.127					R^2^ = 0.344			
		F (2, 207) = 15.029, *p* < 0.001					F (3, 206) = 36.036, *p* < 0.001			

**Table 5:** Model one mediation summary for cognitive control.

Total Effect	Direct Effect	Indirect Effect	Confidence Interval (LB, UB)	*t*-Statistic	Conclusion
0.135 ***	0.100 **	0.035 ***	(0.010, 0.065)	25.286	Partial Mediation

*** *p* < 0.001, ** *p* < 0.01.

**Table 6:** Model two mediation analysis for cognitive flexibility.

		M (Social Isolation)						Y (Life Satisfaction)		
Antecedent		B	SE	*p*	β		B	SE	*p*	β
X (Cognitive flexibility)	a	−0.264	0.047	0.000	−0.364	c′	0.145	0.033	0.000	0.261
M (Social isolation)						b	−0.109	0.046	0	−0.142
		R^2^ = 0.149					R^2^ = 0.377			
		F (2, 207) = 18.130, *p* < 0.001					F (3, 206) = 41.627, *p* < 0.001			

**Table 7:** Model two mediation summary for cognitive flexibility.

Total Effect	Direct Effect	Indirect Effect	Confidence Interval (LB, UB)	*t*-statistic	Conclusion
0.173 ***	0.145 ***	0.029 ***	(0.006, 0.058)	21.103	Partial Mediation

*** *p* < 0.001.

**Table 8:** Combined mediation analysis summary.

	Standardized Direct Effects	Standardized Indirect Effects	Standardized Total Effects	Conclusion
Cognitive flexibility --> Social isolation --> Intrinsic job satisfaction	0.231 *	0.035 *	0.265 ***	Partial Mediation
Cognitive control --> Social isolation --> Intrinsic job satisfaction	0.072	0.027 *	0.1	Full Mediation

*** *p* < 0.001, * *p* < 0.05.

## Data Availability

The data that support the findings of this study are available from the corresponding author upon reasonable request.
